# Supplementary material for: Functional Brain Network Topology Discriminates between Patients with Minimally Conscious State and Unresponsive Wakefulness Syndrome
Source: J Clin Med. 2019 Mar 5;8(3):306. doi: 10.3390/jcm8030306 (PMC6463121; doi:10.3390/jcm8030306)
Supplement: Supplementary file 1 [file jcm-08-00306-s001.pdf]

Supplementary Methods

Stochastic Measures

The *small-worldness* [1,2] was proposed for the characterization of a given network as small-world (SW), meaning that it exhibits a high average clustering coefficient and a low characteristic path length [3]. It relies on comparing a given network with an equivalent random network and lattice network on the basis of the average clustering coefficient, a local measure, and the characteristic path length, a global measure. In 2006 a coefficient called 'σ' for characterizing SW networks was introduced by Humphries et al. [1]. To calculate this measure, the average clustering coefficient C and characteristic path length L of the network are compared to C_{rand} and L_{rand} of an equivalent random network (with similar node degree distribution), obtaining the SW coefficient:

$$\sigma = \frac{C/C_{rand}}{L/L_{rand}} \tag{1}$$

A condition for a network to exhibit small-worldness is that the characteristic path length should be close to that of an equivalent random network, $L \approx L_{rand}$. At the same time, the average clustering coefficient should be close to that of an equivalent lattice network, which also implies that C should be much higher than that of equivalent random network, $C \gg C_{rand}$. These boundary conditions, if met, restrict the value of $\sigma > 1$ for small-world networks. The problem with this coefficient is that even small variations in the already low value of the average clustering coefficient for random networks, C_{rand}, significantly influences the value of the ratio C/C_{rand}. To overcome this problem, a new robust measure was introduced by Telesford et al. [2] which is called 'ω'. The characteristic path length L is compared to L_{rand} of an equivalent random network and the average clustering coefficient C is compared to C_{latt} of an equivalent lattice network, obtaining the SW coefficient:

$$\omega = \frac{L_{rand}}{L} - \frac{C}{C_{latt}} \tag{2}$$

Note that C_{rand} is not considered, therefore this measure neglects its fluctuations. Since the boundary conditions for small-worldness are $L \approx L_{rand}$ and $C \approx C_{latt}$, the values of ω are expected to be close to 0 in small-world networks. The equation suggests that the typical range for the coefficient is $\omega \in [-1,1]$, with positive values representing a network closer to a random one ($L \approx L_{rand}$ and $C \ll C_{latt}$), and negative values representing a network closer to a lattice ($L \gg L_{rand}$ and $C \approx C_{latt}$).

Taking into account the abovementioned limitations for σ, in the present study we decided to calculate the SWω coefficient.

The *modularity* [4,5] is a global measure that indicates the possible presence of segregated modules or communities in a network. In networks with high modularity, the modules tend to interact densely within themselves but sparsely or not at all between each other. The modularity index Q measures the quality of the best possible partition of nodes, which maximizes the intra-module connectivity and minimizes the inter-modules connectivity. It has been introduced according to the following formula [4]:

$$Q = \sum_{u \in M} \left[e_{uu} - \left(\sum_{v \in M} e_{uv} \right)^2 \right] \tag{3}$$

Where M is a partition of the nodes (whose elements u are called modules) and e_{uv} is the proportion of links in module u connecting to module v. If the number of intra-module edges is no better than random, Q is close to 0, whereas values approaching to the maximum Q = 1 indicates strong community structure, although values for such networks typically fall in the range (0.3, 0.7) [4]. Several methods for finding the optimal modularity of a network has been proposed [4–6] and they rely on different heuristics for sampling the partition space [6]. The randomness in the sampling procedure leads to the stochasticity in the measure.

The *structural consistency* [7] is a global measure that quantifies the link predictability of a complex network. The link predictability characterizes the inherent difficulty to predict the missing or non-observed links of a network regardless of the specific algorithm used for the prediction. Its evaluation relies on a random perturbation (which is origin of stochasticity) and first-order approximation of the adjacency matrix. It is based on the hypothesis that a group of links is highly predictable if their addition does not cause huge structural changes, therefore a network is highly predictable if the removal or addition of a set of randomly selected links does not significantly change the network's structural features. [7]. The measure assumes values in the interval [0,1], where 0 indicates absence of link predictability and 1 indicates full link predictability.

Deterministic measures

The *characteristic path length (L)* [2,3] is a global measure and describes the average of the shortest path lengths between all the pairs of vertices. It is defined as:

$$L = \frac{2}{n(n-1)} \sum_{i<j} sp_{ij} \tag{4}$$

Where sp_{ij} is the shortest path length between a pair of nodes $(i,j) \in N$ and $\frac{2}{n(n-1)}$ is the number of possible node pairs in an undirected network. A small value of L in a connectome means that the information flow between the nodes across the network is facilitated, and that the nodes are able to exchange messages between each other easily. In other words, the nodes across connectomes are functionally convergent.

The *average efficiency (E_{glob})* [8,9] is a global measure that quantifies how efficiently the information is exchanged within the network. It is inversely proportional to the *L*, therefore a network with low characteristic path length is highly efficient.

$$E_{glob} = \frac{2}{n(n-1)} \sum_{i<j} \frac{1}{sp_{ij}} \tag{5}$$

Where sp_{ij} is the shortest path length between a pair of nodes $(i,j) \in N$ and $\frac{2}{n(n-1)}$ is the number of possible node pairs in an undirected network.

The *average local efficiency* (E_{loc}) reflects the extent of integration between the immediate neighbors of a given node. In this way, local efficiency can be considered a generalization of the clustering coefficient that explicitly takes into account paths. The clustering coefficient only counts the direct connections between the neighbors of a node, whereas both indirect paths and direct connections are considered by local efficiency. It has been argued that local efficiency also provides a measure of fault tolerance that indicates how efficiently the neighbors of a node are able to communicate when that node is disrupted.

$$E_{loc}(i) = \frac{1}{N_{G_i}(N_{G_i} - 1)} \sum_{j,h \in G_i} \frac{1}{sp_{jh}} \tag{6}$$

where G_i denotes the subgraph comprising all nodes that are immediate neighbors of the ith node.

The *average clustering coefficient (C)* [3] is a local measure and offers an average evaluation of the cross-interaction density between the first neighbours of each node in the network.

$$C = \frac{1}{n} \sum_{i} \frac{2t_i}{d_i(d_i - 1)} \tag{7}$$

Where t_i is the number of cross-interactions that occur between the first neighbours of the node $i \in N$ and $\frac{2}{d_i(d_i-1)}$ is the total number of possible cross-interactions that could occur between them. It assumes values in the range [0,1], large values indicate that the nodes in the network tend to have highly connected neighbours.

The *average node betweeness centrality (NBC)* [10] is a global measure based on the node betweenness centrality, an indicator of node centrality that evaluates how crucial a particular node is

in maintaining a path of optimum information flow between any other pair of nodes. The average measure calculates the average stress of information burden on the network nodes. For a single node it is defined as:

$$NBC_i = \sum_{j \neq i \neq k} \frac{\sigma_{jk}(i)}{\sigma_{jk}} \quad (8)$$

Where $i, j, k \in N$, σ_{jk} is the total number of shortest paths between j and k and $\sigma_{jk}(i)$ is the number of those paths passing through i. *NBC* is obtained as average over the nodes $i \in N$.

In contrast to the existing node-neighbourhood-based local measures, a new strategic shift has been introduced recently in which the focus is no longer only on groups of nodes and their common neighbours, but also on the organization of the links between them [11]. This new idea inspired a theory, which is known as the local community paradigm (LCP-theory) and is valid both in monopartite [11] and in bipartite [12,13] undirected unweighted networks. The *LCP-theory* was proposed to mechanistically and deterministically model local-topology-dependent link-growth in complex networks, and holds that for modelling link prediction in complex networks, the information content related with the common neighbour nodes (CNs) of a given link should be complemented with the topological information emerging from the interactions between them. The cohort of CNs and their cross-interactions—which are called local community links (LCLs)—form what is called a local community. This first part of the theory inspired the Cannistraci variation of the classical CN-based similarity indices for link prediction, named also LCP-based link predictors, for details refers to Cannistraci et al. [11–13].

Furthermore, the LCP-theory holds that in many complex network topologies, the number of CNs of each link in the network is positively correlated with the respective number of LCLs. This second part of the LCP-theory motivated a new network measure called *local-community-paradigm correlation (LCP-corr)* [11–13], which is a local measure that represents an exception with respect to the majority of the previous ones, for two main reasons. Firstly, it is not related with only the node neighbourhood but with the node/link neighbourhood. Secondly, the general statistic used to obtain a unique value is not the average but the Pearson correlation. The formula for computing the LCP-corr is:

$$LCPcorr = \frac{cov(CN, LCL)}{\sigma_{CN} \cdot \sigma_{LCL}}, when\ CN > 0 \quad (9)$$

where *cov* indicates the covariance operator and σ the standard deviation. This formula is clearly a Pearson correlation between the CN and LCL variables. CN indicates a one-dimensional array. Its length is equal to the number of links in the network that have at least one common neighbour, and it reports the number of common neighbours for each of them. LCL indicates a one-dimensional array of the same size as CN, and it reports the number of local community links between the common neighbours. Mathematically the value of LCP-corr would be in the interval [−1,1]. But, extensive tests on many artificial and real complex networks demonstrate that an inverse correlation between CN and LCL is unlikely, therefore the interval is in general between [0,1]. In particular, it was revealed that LCP networks (with high LCP-corr, i.e., > 0.7) are very frequent to occur, and they are related to dynamic and heterogeneous systems that are characterised by weak interactions (relatively expensive or relatively strong) that in turn facilitate network evolution and remodelling. These are typical features of social and biological systems, where the LCP architecture facilitates not only the rapid delivery of information across the various network modules, but also the local processing. In contrast, non-LCP networks (with low LCP-corr, i.e., < 0.4) are less frequent to occur and characterise steady and homogeneous systems that are assembled through strong (often quite expensive) interactions, difficult to erase. This non-LCP architecture is more useful for processes where: (i) the storage of information (or energy) is at least as important as its delivery; (ii) the cost of creating new interactions is excessive; (iii) the creation of a redundant and densely connected system is strategically inadvisable. An emblematic example is the road networks, for which the money and time costs of creating additional roads are very high, and in which a community of strongly connected and crowded links resembles an impractical labyrinth.

In normal conditions, brain connectomes follow LCP organization [11], therefore they are characterized by high LCP-corr, which is in general higher than 0.8.

Table S1. List of the fifty-four edges connecting thirty-two different cortical areas in the subnetwork of decreased functional connectivity in UWS patients compared to the MCS patients in the β1 frequency.

REGION A	REGION B	T-STAT
L-BA8	L-BA23	3.30
L-BA31	L-BA32	2.63
L-BA23	L-BA33	3.08
L-BA11	R-BA1	2.93
L-BA32	R-BA1	3.03
L-BA32	R-BA2	4.08
L-BA33	R-BA2	3.43
L-BA10	R-BA3	3.07
L-BA32	R-BA3	3.03
L-BA9	R-BA4	2.95
L-BA10	R-BA4	2.62
L-BA33	R-BA4	2.62
L-BA9	R-BA5	2.67
L-BA10	R-BA5	3.25
L-BA11	R-BA5	3.13
L-BA45	R-BA5	2.64
L-BA47	R-BA5	3.33
L-BA9	R-BA6	2.82
L-BA32	R-BA6	3.51
L-BA9	R-BA7	2.71
L-BA10	R-BA7	2.63
L-BA11	R-BA7	3.87
L-BA33	R-BA7	3.19
L-BA45	R-BA7	2.89
L-BA46	R-BA7	3.07
L-BA47	R-BA7	3.01
L-BA9	R-BA13	2.70
L-BA8	R-BA23	2.86
L-BA9	R-BA23	3.32
L-BA32	R-BA23	2.80
L-BA33	R-BA23	3.26
R-BA17	R-BA25	3.01
R-BA23	R-BA25	2.74
L-BA8	R-BA31	2.97
L-BA9	R-BA31	2.94
L-BA11	R-BA31	4.67
L-BA25	R-BA31	2.64
L-BA32	R-BA31	2.71
L-BA33	R-BA31	2.61
R-BA25	R-BA31	2.62
R-BA1	R-BA32	3.06
R-BA5	R-BA32	3.37
R-BA6	R-BA32	2.91
R-BA31	R-BA32	3.08
R-BA19	R-BA33	2.84
R-BA31	R-BA33	2.74
L-BA11	R-BA39	3.06
L-BA9	R-BA40	2.61
L-BA32	R-BA40	3.12
L-BA33	R-BA40	2.61
R-BA33	R-BA40	3.02
L-BA9	R-BA41	2.61

L-BA9	R-BA43	3.02
R-BA33	R-BA44	2.62

p = 0.004, corrected for multiple comparison.

Table S2. Clinical-electrophysiological correlation coefficients for every single edge belonging to the dysconnectivity subnetwork identified in UWS by the NBS analysis.

Edge	Pearson Rho	Pearson *p*-value	Edge	Spearman Rho	Spearman *p*-value
L_BA47-R_BA7	0.53	**0.007**	L_BA47-R_BA7	0.54	**0.006**
L_BA33-R_BA2	0.51	**0.009**	L_BA47-R_BA5	0.51	**0.009**
L_BA9-R_BA43	0.51	**0.010**	L_BA46-R_BA7	0.51	**0.010**
R_BA33-R_BA44	0.49	**0.014**	L_BA33-R_BA40	0.50	**0.010**
L_BA33-R_BA4	0.48	**0.014**	R_BA33-R_BA44	0.49	**0.012**
L_BA33-R_BA40	0.48	**0.015**	L_BA23-L_BA33	0.48	**0.015**
L_BA11-R_BA31	0.48	**0.016**	L_BA33-R_BA2	0.47	**0.017**
L_BA46-R_BA7	0.46	**0.021**	L_BA11-R_BA31	0.46	**0.019**
L_BA32-R_BA2	0.45	**0.026**	L_BA33-R_BA7	0.44	**0.027**
L_BA23-L_BA33	0.44	**0.027**	L_BA33-R_BA4	0.44	**0.027**
L_BA10-R_BA3	0.43	**0.034**	L_BA9-R_BA43	0.44	**0.028**
L_BA47-R_BA5	0.42	**0.036**	R_BA5-R_BA32	0.43	**0.031**
L_BA10-R_BA5	0.42	**0.037**	L_BA11-R_BA5	0.43	**0.032**
L_BA11-R_BA39	0.41	**0.039**	L_BA10-R_BA5	0.43	**0.033**
L_BA45-R_BA7	0.41	**0.039**	L_BA10-R_BA7	0.42	**0.035**
L_BA9-R_BA4	0.41	**0.039**	L_BA45-R_BA7	0.42	**0.036**
L_BA11-R_BA7	0.40	**0.048**	L_BA9-R_BA23	0.42	**0.039**
L_BA33-R_BA7	0.40	**0.048**	L_BA8-L_BA23	0.41	**0.041**
L_BA8-L_BA23	0.39	0.051	R_BA6-R_BA32	0.40	**0.047**
L_BA9-R_BA40	0.39	0.054	L_BA32-R_BA2	0.40	**0.049**
R_BA25-R_BA31	0.36	0.07	L_BA11-R_BA39	0.39	0.051
L_BA11-R_BA5	0.36	0.08	L_BA33-R_BA23	0.38	0.06
R_BA5-R_BA32	0.36	0.08	R_BA31-R_BA32	0.38	0.06
L_BA33-R_BA23	0.36	0.08	L_BA45-R_BA5	0.37	0.07
L_BA9-R_BA6	0.36	0.08	L_BA10-R_BA3	0.37	0.07
R_BA33-R_BA40	0.36	0.08	L_BA32-R_BA6	0.36	0.08
L_BA32-R_BA6	0.35	0.08	R_BA33-R_BA40	0.36	0.08
R_BA1-R_BA32	0.35	0.09	R_BA1-R_BA32	0.34	0.09
R_BA6-R_BA32	0.35	0.09	L_BA9-R_BA4	0.34	0.09
L_BA9-R_BA23	0.34	0.09	R_BA25-R_BA31	0.34	0.10
L_BA32-R_BA1	0.34	0.10	L_BA9-R_BA6	0.33	0.11
R_BA17-R_BA25	0.33	0.10	L_BA11-R_BA7	0.33	0.11
L_BA10-R_BA7	0.33	0.11	L_BA9-R_BA40	0.33	0.11
R_BA31-R_BA32	0.33	0.11	L_BA32-R_BA40	0.32	0.12
R_BA23-R_BA25	0.32	0.12	L_BA32-R_BA1	0.31	0.13
L_BA32-R_BA40	0.32	0.12	L_BA11-R_BA1	0.31	0.13
L_BA9-R_BA5	0.31	0.14	R_BA23-R_BA25	0.30	0.15
L_BA9-R_BA41	0.30	0.14	L_BA25-R_BA31	0.29	0.16
L_BA32-R_BA3	0.30	0.14	L_BA9-R_BA41	0.28	0.17
L_BA10-R_BA4	0.29	0.15	L_BA32-R_BA3	0.28	0.17
L_BA9-R_BA7	0.29	0.16	L_BA9-R_BA5	0.28	0.17
L_BA11-R_BA1	0.28	0.18	R_BA19-R_BA33	0.28	0.18
L_BA31-L_BA32	0.26	0.21	L_BA8-R_BA23	0.27	0.19
L_BA8-R_BA23	0.26	0.22	R_BA17-R_BA25	0.26	0.22
L_BA32-R_BA31	0.26	0.22	L_BA9-R_BA7	0.25	0.23
L_BA45-R_BA5	0.26	0.22	L_BA32-R_BA31	0.25	0.23
R_BA19-R_BA33	0.25	0.22	R_BA31-R_BA33	0.25	0.23
L_BA9-R_BA31	0.24	0.24	L_BA9-R_BA13	0.24	0.25
L_BA9-R_BA13	0.23	0.27	L_BA32-R_BA23	0.24	0.25
L_BA8-R_BA31	0.22	0.29	L_BA33-R_BA31	0.23	0.28
L_BA25-R_BA31	0.20	0.33	L_BA31-L_BA32	0.22	0.28
L_BA32-R_BA23	0.20	0.33	L_BA10-R_BA4	0.22	0.28

R_BA31-R_BA33	0.18	0.39	L_BA9-R_BA31	0.20	0.35
L_BA33-R_BA31	0.15	0.49	L_BA8-R_BA31	0.10	0.63

The table reports both the Pearson's and Spearman's rho and related *p*-values. Statistically significant correlations ($p < 0.05$) between the edge strength and the Coma Recovery Scale-Revised scores have been highlighted in bold character.

Table S3. Single node topological measures differences between UWS and MCS.

	Degree		*Betweeness Centrality*		*Clustering Coefficient*	
**Node Label**	**MW *p*-value**	**BH-corrected *p*-value**	**MW *p*-value**	**BH-corrected *p*-value**	**MW *p*-value**	**BH-corrected *p*-value**
**L-BA1**	0.429	0.819	0.744	0.947	0.173	0.502
**L-BA2**	0.643	0.916	0.532	0.877	0.744	0.906
**L-BA3**	0.165	0.629	0.220	0.740	1.000	1.000
**L-BA4**	0.091	0.573	0.087	0.455	0.586	0.858
**L-BA5**	**0.010**	0.399	**0.047**	0.395	0.092	0.405
**L-BA6**	0.313	0.764	0.430	0.877	0.605	0.858
**L-BA7**	0.892	0.968	0.724	0.935	**0.041**	0.248
**L-BA8**	0.157	0.627	0.265	0.742	0.135	0.471
**L-BA9**	**0.038**	0.402	**0.032**	0.380	0.183	0.511
**L-BA10**	0.102	0.573	**0.047**	0.395	**0.005**	0.232
**L-BA11**	**0.015**	0.399	0.201	0.704	0.253	0.574
**L-BA13**	0.849	0.968	0.978	1.000	0.550	0.851
**L-BA17**	0.141	0.627	**0.024**	0.380	0.265	0.574
**L-BA18**	**0.020**	0.399	**0.007**	0.350	0.531	0.851
**L-BA19**	**0.046**	0.432	**0.032**	0.380	0.149	0.472
**L-BA20**	0.479	0.833	0.314	0.776	0.157	0.472
**L-BA21**	0.723	0.964	0.463	0.877	0.414	0.722
**L-BA22**	0.477	0.833	0.683	0.926	1.000	1.000
**L-BA23**	0.586	0.895	0.341	0.796	0.121	0.462
**L-BA24**	0.870	0.968	0.849	0.977	0.230	0.553
**L-BA25**	0.355	0.764	0.849	0.977	**0.010**	0.232
**L-BA27**	0.764	0.968	0.935	0.982	0.127	0.465
**L-BA28**	0.531	0.875	0.605	0.877	0.644	0.858
**L-BA29**	0.210	0.705	0.369	0.817	**0.039**	0.248
**L-BA30**	0.251	0.707	0.165	0.697	**0.014**	0.232
**L-BA31**	0.892	0.968	0.605	0.877	0.221	0.546
**L-BA32**	0.068	0.505	0.183	0.697	**0.036**	0.248
**L-BA33**	**0.024**	0.399	**0.008**	0.350	0.068	0.319
**L-BA34**	0.935	0.968	0.683	0.926	**0.008**	0.232
**L-BA35**	0.827	0.968	0.807	0.977	0.314	0.574
**L-BA36**	0.219	0.707	0.532	0.877	0.157	0.472
**L-BA37**	0.156	0.627	0.053	0.395	0.314	0.574
**L-BA38**	0.446	0.832	0.341	0.796	0.068	0.319
**L-BA39**	0.252	0.707	0.183	0.697	**0.021**	0.232
**L-BA40**	0.340	0.764	0.430	0.877	0.624	0.858
**L-BA41**	0.413	0.807	0.605	0.877	0.624	0.858
**L-BA42**	0.957	0.968	0.807	0.977	0.430	0.722
**L-BA43**	0.353	0.764	0.289	0.758	0.892	1.000
**L-BA44**	1.000	1.000	0.568	0.877	0.935	1.000
**L-BA45**	0.703	0.964	0.683	0.926	**0.036**	0.248
**L-BA46**	**0.038**	0.402	**0.021**	0.380	**0.021**	0.232
**L-BA47**	0.134	0.627	0.242	0.742	0.121	0.462
**R-BA1**	0.567	0.895	0.935	0.982	1.000	1.000
**R-BA2**	0.935	0.968	0.532	0.877	0.703	0.882
**R-BA3**	0.892	0.968	0.935	0.982	0.644	0.858
**R-BA4**	0.828	0.968	0.568	0.877	0.549	0.851
**R-BA5**	0.764	0.968	0.644	0.917	0.289	0.574
**R-BA6**	0.384	0.786	0.115	0.535	0.957	1.000
**R-BA7**	0.060	0.505	0.053	0.395	0.221	0.546
**R-BA8**	0.253	0.707	0.242	0.742	0.341	0.609
**R-BA9**	0.102	0.573	0.265	0.742	0.288	0.574
**R-BA10**	0.785	0.968	0.724	0.935	**0.034**	0.248

**R-BA11**	0.191	0.699	0.183	0.697	0.935	1.000
**R-BA13**	0.585	0.895	0.369	0.817	0.568	0.851
**R-BA17**	0.462	0.833	0.568	0.877	0.053	0.300
**R-BA18**	0.287	0.764	0.201	0.704	0.157	0.472
**R-BA19**	0.156	0.627	0.077	0.455	0.221	0.546
**R-BA20**	0.956	0.968	0.892	0.982	0.891	1.000
**R-BA21**	0.723	0.964	0.978	1.000	0.828	0.965
**R-BA22**	0.327	0.764	0.497	0.877	0.957	1.000
**R-BA23**	0.956	0.968	0.724	0.935	**0.041**	0.248
**R-BA24**	0.827	0.968	1.000	1.000	0.786	0.929
**R-BA25**	0.368	0.774	0.265	0.742	0.289	0.574
**R-BA27**	0.624	0.916	0.870	0.982	0.683	0.882
**R-BA28**	0.935	0.968	0.605	0.877	0.978	1.000
**R-BA29**	**0.034**	0.402	0.097	0.480	0.289	0.574
**R-BA30**	0.072	0.505	0.068	0.442	0.068	0.319
**R-BA31**	0.643	0.916	0.605	0.877	0.314	0.574
**R-BA32**	0.327	0.764	0.849	0.977	0.221	0.546
**R-BA33**	**0.021**	0.399	**0.028**	0.380	0.121	0.462
**R-BA34**	0.913	0.968	0.605	0.877	0.913	1.000
**R-BA35**	0.127	0.627	0.087	0.455	0.957	1.000
**R-BA36**	0.313	0.764	0.056	0.395	0.566	0.851
**R-BA37**	0.253	0.707	0.497	0.877	0.314	0.574
**R-BA38**	0.413	0.807	0.495	0.877	0.682	0.882
**R-BA39**	0.495	0.833	1.000	1.000	0.301	0.574
**R-BA40**	0.785	0.968	0.314	0.776	0.430	0.722
**R-BA41**	0.354	0.764	0.586	0.877	**0.018**	0.232
**R-BA42**	0.209	0.705	0.496	0.877	**0.022**	0.232
**R-BA43**	0.682	0.955	0.891	0.982	0.461	0.759
**R-BA44**	0.605	0.907	0.288	0.758	0.701	0.882
**R-BA45**	0.496	0.833	0.913	0.982	0.643	0.858
**R-BA46**	0.586	0.895	0.806	0.977	0.764	0.917
**R-BA47**	0.827	0.968	0.849	0.977	0.743	0.906

The table reports for each measure (degree, betweenness centrality and clustering coefficient) and for each single network node the Mann–Whitney (MW) *p*-values and the Benjamini-Hochberg (BH) corrected *p*-values. Node that significant *p*-values are highlighted in bold, considering a confidence level of 0.05. It is worthy to note that the fact that the correction for multiple hypothesis testing nullifies any evidence of significant *p*-values does not mean that this is strictly true, indeed this just suggests that the results obtained without correction should be considered with a grain of salt and caution. These findings offer an indication that needs further investigation, but at the moment they cannot be considered as markers to discriminate between UWS and MCS.

Table S4. Clinical-topological (degree) correlation coefficients for every single network node.

Node	Pearson Rho	Pearson *p*-value	Node	Spearman Rho	Spearman *p*-value
L-BA5	−0.58	**0.002**	L-BA5	−0.61	**0.001**
L-BA46	0.50	**0.010**	L-BA37	−0.46	**0.022**
L-BA47	0.48	**0.015**	L-BA47	0.43	**0.033**
L-BA37	−0.45	**0.025**	L-BA40	−0.43	**0.033**
R-BA33	0.44	**0.029**	L-BA46	0.42	**0.036**
L-BA33	0.41	**0.043**	R-BA33	0.41	**0.041**
L-BA10	0.36	0.08	L-BA41	−0.41	**0.043**
L-BA41	−0.35	0.08	L-BA3	−0.40	**0.046**
L-BA3	−0.35	0.09	L-BA4	−0.38	0.06
L-BA43	0.34	0.10	L-BA10	0.36	0.08
L-BA22	−0.32	0.12	L-BA33	0.35	0.09
L-BA40	−0.31	0.13	L-BA11	0.34	0.09
R-BA9	0.31	0.13	L-BA31	−0.31	0.13
R-BA8	0.30	0.15	L-BA22	−0.31	0.14
L-BA31	−0.29	0.16	L-BA39	0.28	0.18
L-BA24	−0.28	0.18	R-BA8	0.28	0.18
R-BA30	−0.27	0.18	R-BA35	0.27	0.19
L-BA11	0.27	0.19	R-BA30	−0.26	0.21
R-BA35	0.27	0.19	L-BA1	−0.26	0.21
R-BA36	0.27	0.20	L-BA34	−0.26	0.21
L-BA4	−0.26	0.21	L-BA36	−0.25	0.22
R-BA29	−0.26	0.22	R-BA28	0.25	0.23
L-BA36	−0.25	0.22	R-BA22	−0.25	0.23
L-BA7	−0.25	0.23	R-BA7	0.23	0.27
L-BA45	0.23	0.27	L-BA27	0.23	0.27
L-BA6	−0.22	0.28	L-BA43	0.23	0.27
L-BA9	0.21	0.31	R-BA29	−0.23	0.28
L-BA34	−0.20	0.33	L-BA24	−0.22	0.28
R-BA22	−0.20	0.34	R-BA9	0.22	0.29
R-BA7	0.20	0.34	R-BA44	0.21	0.32
L-BA39	0.20	0.34	L-BA6	−0.20	0.33
R-BA19	−0.20	0.35	R-BA36	0.20	0.33
R-BA37	−0.19	0.36	L-BA35	0.18	0.39
R-BA38	−0.19	0.36	L-BA20	−0.17	0.41
R-BA44	0.19	0.36	L-BA21	0.17	0.41
L-BA8	0.19	0.38	R-BA19	−0.17	0.42
R-BA43	0.18	0.38	R-BA11	0.16	0.43
R-BA24	−0.17	0.43	L-BA42	−0.16	0.45
R-BA28	0.17	0.43	R-BA43	0.15	0.46
R-BA21	−0.16	0.44	R-BA41	−0.15	0.48
L-BA1	−0.16	0.44	R-BA21	−0.14	0.49
L-BA21	0.15	0.46	L-BA8	0.14	0.50
L-BA38	0.14	0.49	R-BA42	−0.14	0.50
R-BA1	0.13	0.53	R-BA46	0.13	0.52
L-BA42	−0.13	0.53	R-BA45	0.13	0.53
R-BA41	−0.12	0.56	R-BA40	−0.13	0.54
L-BA35	0.12	0.57	L-BA9	0.12	0.56
L-BA23	0.11	0.59	L-BA18	−0.12	0.57
L-BA20	−0.11	0.59	R-BA32	−0.12	0.57
R-BA25	0.11	0.61	R-BA23	−0.12	0.57
L-BA13	−0.11	0.61	R-BA1	0.12	0.57
R-BA46	0.10	0.62	R-BA31	−0.11	0.59
L-BA18	−0.10	0.65	R-BA34	0.11	0.60

R-BA32	−0.09	0.66	R-BA38	−0.11	0.60
L-BA19	−0.09	0.67	R-BA47	0.11	0.61
L-BA28	0.09	0.68	R-BA27	0.11	0.61
R-BA3	0.08	0.69	L-BA23	0.10	0.63
R-BA10	−0.08	0.70	L-BA13	−0.10	0.63
L-BA44	−0.08	0.71	L-BA7	−0.10	0.64
L-BA27	0.08	0.71	L-BA28	−0.10	0.64
L-BA25	0.08	0.71	L-BA25	0.09	0.66
R-BA23	−0.08	0.72	R-BA10	−0.08	0.70
R-BA11	0.08	0.72	L-BA32	0.08	0.71
R-BA5	−0.07	0.74	R-BA37	−0.08	0.71
L-BA32	0.07	0.76	L-BA44	−0.08	0.71
R-BA2	0.06	0.77	R-BA17	0.08	0.72
R-BA40	−0.06	0.77	R-BA4	−0.07	0.73
R-BA42	−0.06	0.77	L-BA45	0.07	0.75
R-BA39	−0.06	0.79	L-BA19	−0.05	0.80
R-BA13	−0.05	0.82	R-BA5	−0.05	0.81
R-BA47	0.05	0.82	L-BA38	0.05	0.81
R-BA34	0.05	0.83	R-BA25	0.05	0.83
R-BA45	−0.03	0.90	R-BA24	−0.04	0.84
R-BA4	0.03	0.90	L-BA30	0.04	0.84
L-BA17	−0.03	0.90	L-BA2	−0.04	0.85
L-BA29	−0.02	0.91	R-BA18	−0.04	0.86
R-BA6	0.02	0.92	R-BA6	0.03	0.88
L-BA2	−0.02	0.93	L-BA29	−0.02	0.92
R-BA18	−0.02	0.93	R-BA2	0.01	0.95
R-BA20	0.01	0.94	R-BA39	0.01	0.96
L-BA30	−0.01	0.95	R-BA3	−0.01	0.96
R-BA27	0.01	0.96	R-BA20	−0.01	0.96
R-BA31	−0.01	0.98	L-BA17	0.01	0.97
R-BA17	0.00	0.99	R-BA13	−0.01	0.98

The table reports both the Pearson's and Spearman's rho and related *p*-values. Statistically significant correlations ($p < 0.05$) between the node degree and the Coma Recovery Scale-Revised scores have been highlighted in bold character.

Table S5. Clinical-topological (betweenness centrality) correlation coefficients for every single network node.

Node	Pearson Rho	Pearson *p*-value	Node	Spearman Rho	Spearman *p*-value
L-BA46	0.51	**0.009**	L-BA37	−0.50	**0.010**
L-BA10	0.50	**0.011**	L-BA5	−0.47	**0.018**
R-BA35	0.48	**0.014**	L-BA46	0.46	**0.021**
L-BA39	0.46	**0.021**	L-BA10	0.44	**0.029**
R-BA36	0.45	**0.023**	L-BA31	−0.43	**0.033**
L-BA31	−0.42	**0.038**	L-BA4	−0.41	**0.042**
L-BA37	−0.40	0.050	L-BA3	−0.40	**0.047**
R-BA33	0.39	0.06	L-BA47	0.38	0.06
R-BA9	0.38	0.06	R-BA35	0.37	0.07
L-BA47	0.38	0.06	L-BA40	−0.36	0.08
L-BA43	0.38	0.06	R-BA30	−0.35	0.08
L-BA33	0.35	0.09	R-BA44	0.35	0.08
L-BA5	−0.34	0.09	L-BA34	−0.35	0.09
L-BA3	−0.31	0.13	R-BA36	0.35	0.09
L-BA24	−0.31	0.13	L-BA27	0.34	0.09
R-BA30	−0.30	0.15	R-BA33	0.33	0.11
R-BA44	0.30	0.15	R-BA8	0.32	0.11
R-BA19	−0.29	0.16	L-BA39	0.30	0.14
R-BA8	0.26	0.20	L-BA33	0.30	0.15
L-BA34	−0.26	0.22	L-BA41	−0.29	0.16
R-BA32	−0.25	0.23	R-BA7	0.29	0.16
L-BA40	−0.25	0.23	R-BA32	−0.26	0.20
R-BA29	−0.24	0.24	R-BA18	−0.24	0.24
L-BA21	0.23	0.27	R-BA19	−0.24	0.24
L-BA8	0.22	0.28	R-BA29	−0.24	0.25
R-BA22	−0.22	0.29	L-BA20	−0.23	0.27
L-BA36	−0.21	0.30	R-BA22	−0.22	0.28
L-BA44	−0.21	0.31	L-BA24	−0.22	0.29
L-BA9	0.21	0.31	L-BA44	−0.22	0.30
L-BA17	−0.21	0.31	L-BA11	0.21	0.31
L-BA1	−0.21	0.32	R-BA11	0.20	0.34
L-BA6	−0.18	0.38	L-BA22	−0.19	0.36
L-BA45	0.17	0.41	R-BA4	−0.19	0.36
R-BA24	−0.17	0.42	L-BA21	0.18	0.39
R-BA7	0.17	0.42	R-BA28	0.18	0.39
R-BA23	−0.16	0.43	L-BA1	−0.16	0.44
L-BA7	−0.16	0.44	L-BA23	0.16	0.46
R-BA43	0.16	0.44	L-BA35	0.15	0.46
R-BA38	−0.15	0.46	L-BA18	−0.14	0.50
R-BA39	−0.15	0.47	R-BA9	0.13	0.52
L-BA23	0.15	0.48	L-BA36	−0.13	0.53
R-BA45	−0.14	0.49	L-BA43	0.13	0.53
L-BA18	−0.14	0.51	L-BA9	0.13	0.55
L-BA11	0.14	0.51	L-BA7	−0.13	0.55
L-BA2	0.14	0.51	L-BA38	0.12	0.56
R-BA4	0.13	0.52	R-BA43	0.11	0.59
R-BA31	−0.13	0.54	R-BA40	−0.11	0.59
R-BA37	−0.12	0.55	R-BA46	0.11	0.60
R-BA41	−0.12	0.57	R-BA6	−0.11	0.62
R-BA42	−0.11	0.59	L-BA32	−0.10	0.64
R-BA46	0.11	0.61	L-BA8	0.10	0.64
L-BA32	−0.11	0.61	R-BA38	−0.10	0.65

R-BA21	−0.10	0.62	R-BA1	0.09	0.67
R-BA34	−0.10	0.63	L-BA19	−0.09	0.68
L-BA38	0.10	0.63	L-BA28	−0.08	0.69
L-BA4	−0.10	0.63	R-BA10	−0.08	0.69
L-BA22	−0.10	0.63	L-BA17	−0.08	0.70
R-BA20	−0.10	0.64	R-BA21	0.08	0.70
R-BA17	−0.10	0.65	R-BA27	0.07	0.73
L-BA20	0.09	0.65	R-BA39	−0.07	0.74
R-BA3	0.09	0.65	R-BA47	0.07	0.75
R-BA25	0.09	0.66	R-BA31	−0.06	0.76
L-BA28	0.08	0.70	L-BA25	−0.06	0.76
R-BA13	−0.07	0.75	L-BA6	−0.06	0.77
R-BA47	0.06	0.79	R-BA23	−0.06	0.77
R-BA18	−0.05	0.79	R-BA41	−0.06	0.77
L-BA29	0.05	0.80	R-BA13	−0.05	0.80
L-BA35	0.05	0.80	R-BA37	−0.05	0.83
L-BA27	0.05	0.81	R-BA25	0.05	0.83
L-BA41	−0.05	0.82	R-BA17	0.05	0.83
L-BA25	−0.05	0.83	R-BA42	−0.04	0.85
L-BA19	0.04	0.84	R-BA2	−0.04	0.85
L-BA42	0.04	0.84	L-BA30	−0.04	0.86
R-BA10	0.04	0.85	L-BA13	−0.03	0.87
R-BA5	0.04	0.86	R-BA3	−0.03	0.87
R-BA27	−0.03	0.87	L-BA29	0.03	0.88
R-BA11	−0.03	0.88	R-BA5	−0.02	0.92
R-BA40	0.03	0.89	L-BA42	−0.02	0.93
L-BA13	0.02	0.91	L-BA2	−0.02	0.93
R-BA28	0.02	0.92	R-BA45	0.02	0.94
R-BA1	0.02	0.94	R-BA20	0.02	0.94
R-BA2	−0.01	0.94	L-BA45	−0.02	0.94
R-BA6	−0.01	0.96	R-BA34	−0.01	0.96
L-BA30	−0.01	0.97	R-BA24	−0.01	0.97

The table reports both the Pearson's and Spearman's rho and related *p*-values. Statistically significant correlations ($p < 0.05$) between the node betweenness centrality and the Coma Recovery Scale-Revised scores have been highlighted in bold character.

Table S6. Clinical-topological (clustering coefficient) correlation coefficients for every single network node.

Node	Pearson Rho	Pearson *p*-value	Node	Spearman Rho	Spearman *p*-value
R-BA41	−0.51	0.009	R-BA41	−0.56	0.003
R-BA42	−0.44	0.026	R-BA42	−0.49	0.012
L-BA21	−0.38	0.06	L-BA21	−0.46	0.021
L-BA10	−0.37	0.07	L-BA36	−0.39	0.052
R-BA47	−0.35	0.08	L-BA37	−0.39	0.054
R-BA6	−0.35	0.08	L-BA10	−0.39	0.055
L-BA39	−0.34	0.09	L-BA39	−0.37	0.07
R-BA46	−0.34	0.09	R-BA46	−0.33	0.10
L-BA36	−0.33	0.11	L-BA24	−0.33	0.11
L-BA37	−0.33	0.11	L-BA7	−0.31	0.13
L-BA34	−0.31	0.13	R-BA47	−0.31	0.13
L-BA25	−0.30	0.14	L-BA30	−0.30	0.14
L-BA5	−0.29	0.16	R-BA10	−0.29	0.16
L-BA28	−0.29	0.16	L-BA25	−0.28	0.18
L-BA35	−0.29	0.17	L-BA35	−0.28	0.18
L-BA24	−0.28	0.17	L-BA34	−0.27	0.20
R-BA4	0.28	0.17	R-BA6	−0.25	0.22
R-BA39	−0.27	0.19	L-BA38	−0.25	0.24
L-BA46	−0.27	0.20	R-BA40	−0.24	0.26
L-BA30	−0.25	0.22	L-BA1	−0.24	0.26
R-BA38	−0.25	0.23	L-BA5	−0.23	0.26
L-BA38	−0.25	0.23	R-BA39	−0.23	0.27
R-BA28	−0.24	0.24	R-BA17	−0.23	0.28
R-BA40	−0.24	0.24	L-BA41	−0.22	0.29
L-BA45	−0.24	0.25	R-BA37	−0.21	0.31
L-BA7	−0.24	0.26	L-BA46	−0.21	0.31
R-BA45	−0.22	0.30	L-BA45	−0.21	0.31
R-BA36	−0.20	0.33	L-BA29	−0.20	0.33
L-BA27	−0.20	0.33	L-BA42	−0.20	0.34
R-BA23	−0.20	0.33	L-BA17	−0.20	0.34
R-BA21	−0.20	0.33	R-BA45	−0.19	0.37
R-BA3	0.20	0.34	R-BA19	−0.19	0.37
L-BA17	−0.18	0.38	L-BA18	−0.18	0.38
L-BA11	−0.18	0.39	L-BA8	−0.18	0.38
L-BA1	−0.17	0.40	L-BA31	−0.17	0.41
R-BA13	0.17	0.40	L-BA9	−0.17	0.42
R-BA10	−0.17	0.41	R-BA38	−0.16	0.44
R-BA5	−0.17	0.41	R-BA28	−0.16	0.44
L-BA41	−0.16	0.44	L-BA4	−0.16	0.45
L-BA47	−0.16	0.46	R-BA36	−0.16	0.45
L-BA19	−0.15	0.46	R-BA11	0.15	0.46
L-BA18	−0.15	0.48	L-BA28	−0.15	0.47
L-BA22	−0.14	0.49	L-BA33	−0.15	0.48
R-BA37	−0.14	0.51	L-BA40	−0.14	0.49
L-BA20	0.13	0.53	R-BA3	0.14	0.50
R-BA29	0.13	0.54	R-BA4	0.14	0.50
R-BA9	−0.12	0.56	L-BA22	−0.14	0.51
R-BA7	−0.12	0.57	R-BA35	0.14	0.51
R-BA27	0.12	0.57	L-BA27	−0.14	0.52
L-BA8	−0.11	0.59	R-BA1	0.13	0.53
L-BA13	0.11	0.60	L-BA2	0.13	0.54
R-BA24	−0.10	0.62	L-BA6	−0.13	0.55
L-BA29	−0.10	0.63	R-BA18	−0.12	0.56

R-BA11	0.10	0.64	L-BA19	−0.12	0.57
L-BA2	0.10	0.65	R-BA22	−0.11	0.59
R-BA22	−0.09	0.66	L-BA47	−0.11	0.59
L-BA42	−0.09	0.68	L-BA32	−0.11	0.59
L-BA33	−0.09	0.68	L-BA23	−0.11	0.61
L-BA44	0.09	0.68	R-BA23	−0.11	0.61
R-BA35	0.08	0.69	R-BA21	−0.09	0.66
R-BA25	−0.08	0.69	R-BA5	−0.09	0.67
R-BA34	−0.08	0.70	R-BA2	0.09	0.68
R-BA30	−0.08	0.71	R-BA31	0.08	0.69
R-BA44	−0.08	0.71	L-BA11	−0.08	0.71
L-BA31	−0.08	0.71	R-BA24	−0.08	0.71
R-BA33	−0.08	0.72	L-BA44	0.08	0.72
L-BA9	−0.08	0.72	R-BA13	0.08	0.72
L-BA3	0.07	0.73	L-BA43	0.07	0.73
R-BA18	0.07	0.73	R-BA29	0.07	0.74
L-BA23	−0.07	0.73	L-BA20	0.07	0.76
L-BA32	−0.07	0.74	R-BA27	0.06	0.76
R-BA17	−0.07	0.75	R-BA32	−0.06	0.78
R-BA8	0.06	0.78	R-BA34	−0.06	0.79
R-BA20	−0.06	0.79	R-BA30	−0.05	0.80
R-BA43	−0.05	0.80	R-BA7	−0.05	0.81
R-BA1	0.05	0.83	R-BA33	−0.05	0.81
L-BA6	−0.04	0.86	R-BA9	−0.05	0.81
R-BA32	0.03	0.87	R-BA25	−0.05	0.82
L-BA4	−0.03	0.87	L-BA3	−0.04	0.84
R-BA2	0.03	0.89	R-BA8	0.02	0.92
L-BA40	−0.02	0.91	L-BA13	−0.02	0.93
R-BA19	−0.01	0.96	R-BA43	−0.02	0.94
L-BA43	−0.01	0.96	R-BA44	−0.01	0.96
R-BA31	0.00	1.00	R-BA20	0.00	0.98

The table reports both the Pearson's and Spearman's rho and related *p*-values. Statistically significant correlations ($p < 0.05$) between the node clustering coefficient and the Coma Recovery Scale-Revised scores have been highlighted in bold character.

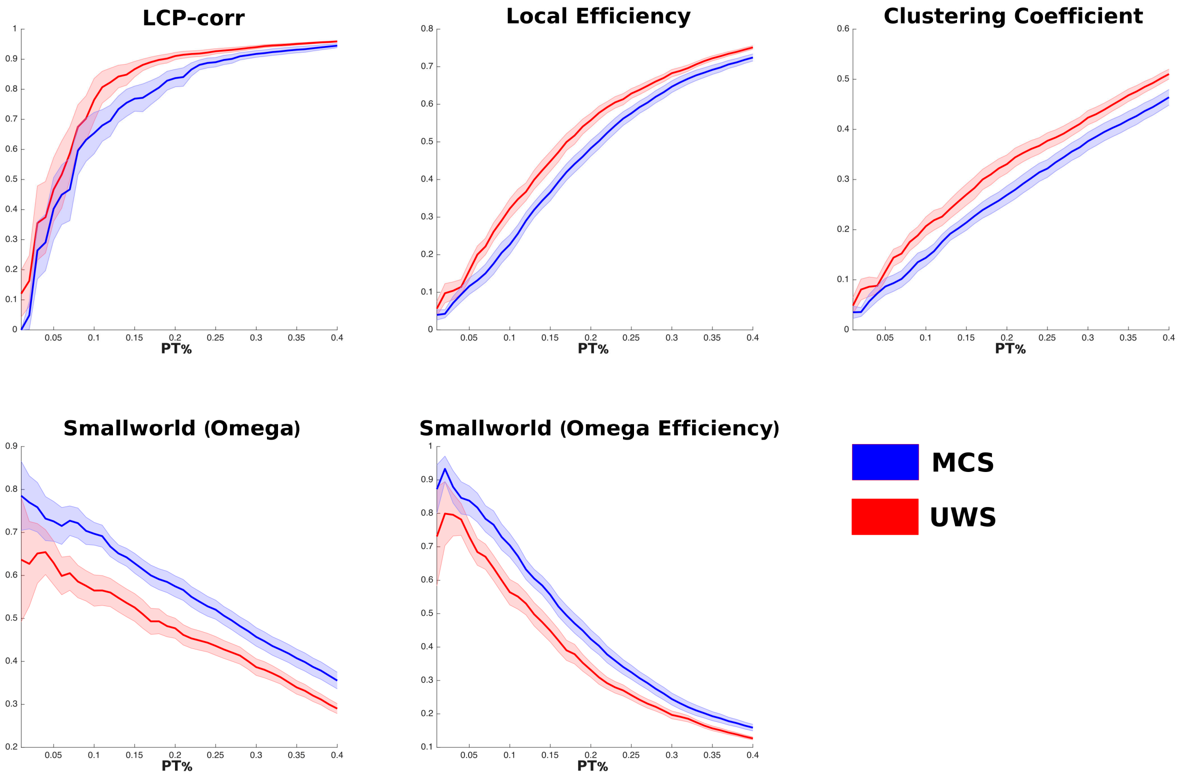

Figure S1. Topological measure curves between UWS and MCS patients across a range of proportional thresholds (35% < PT < 1%). Proportional thresholding was performed on the functional connectivity matrices by selecting the proportional thresholded (PT%) strongest connections of the derived LPS-weighted connectivity matrix and setting these connections to 1, whereas all other connections to 0. Proportional thresholding of a functional connectivity matrix thus resulted in a binary graph with a density of PT%. We examined a range of levels of PT from 35% to 1% in steps of 1% so to depict the trend of the topological measures in UWS and MCS patients across the whole range of thresholds. Considering the robustness of the curves and results, we have then defined to use the specific cut-off of PT 20% to illustrate results, in line with previous works [14,15].

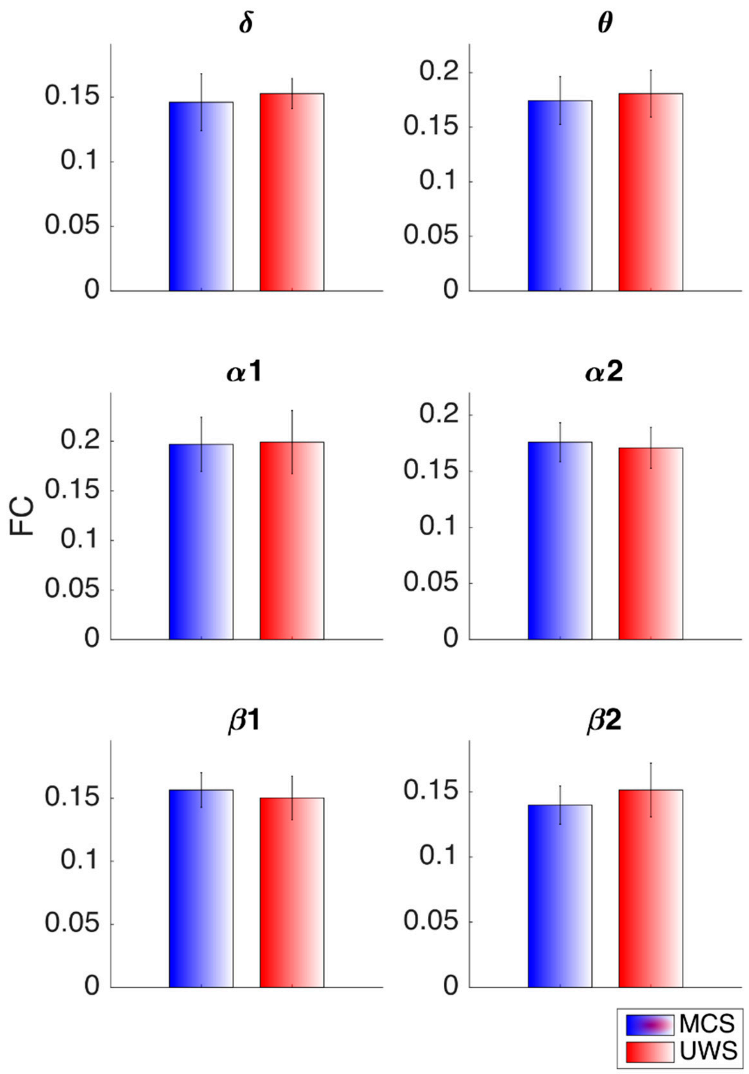

Figure S2. Average overall functional connectivity (FC) strength between the UWS and MCS patients, computed as the mean of the absolute values of the edge weights (strengths) in the connectomes. We performed a Mann-Whitney test to compare (for each of the 6 EEG frequency types) the overall FC in UWS and MCS patients in order to investigate the extent to which a biasing effect for overall FC was present in the data. In this way, it is possible to exclude that the altered topology of a network is dependent from the FC between groups. No statistically significant changes ($p > 0.05$) have been detected between the two groups.

References

1. Humphries, M.D.; Gurney, K. Network "small-world-ness": A quantitative method for determining canonical network equivalence. *PLoS One* **2008**, *3*, e0002051, doi:10.1371/journal.pone.0002051
2. Telesford, Q.K.; Joyce, K.E.; Hayasaka, S.; Burdette, J.H.; Laurienti, P.J. The ubiquity of small-world networks. *Brain Connect.* **2011**, *1*, 367–375.
3. Watts, D.J.; Strogatz, S.H. Collective dynamics of 'small-world' networks. *Nature* **1998**, *393*, 440–442.
4. Newman, M.; Girvan, M. Finding and evaluating community structure in networks. *Phys. Rev. E* **2004**, *69*, 1–16.
5. Newman, M.E.J. Modularity and community structure in networks. *Proc. Natl. Acad. Sci. U. S. A.* **2006**, *103*, 8577–8582.
6. Good, B.H.; De Montjoye, Y.A.; Clauset, A. Performance of modularity maximization in practical contexts. *Phys. Rev. E - Stat. Nonlinear, Soft Matter Phys.* **2010**, *81*, 046106, doi:10.1103/PhysRevE.81.046106.
7. Lü, L.; Pan, L.; Zhou, T.; Zhang, Y.-C.; Stanley, H.E. Toward link predictability of complex networks. *Proc. Natl. Acad. Sci.* **2015**, *112*, 2325–2330.
8. van den Heuvel, M.P.; Stam, C.J.; Kahn, R.S.; Hulshoff Pol, H.E. Efficiency of functional brain networks and intellectual performance. *J. Neurosci.* **2009**, *29*, 7619–7624.
9. Latora, V.; Marchiori, M. Efficient behavior of small-world networks. *Phys. Rev. Lett.* **2001**, *87*, 198701.
10. Brandes, U. A faster algorithm for betweenness centrality. *J. Math. Sociol.* **2001**, *25*, 163–177.
11. Cannistraci, C.V.; Alanis-Lobato, G.; Ravasi, T. From link-prediction in brain connectomes and protein interactomes to the local-community-paradigm in complex networks. *Sci. Rep.* **2013**, *3*, 1–13.
12. Daminelli, S.; Thomas, J.M.; Durán, C.; Vittorio Cannistraci, C. Common neighbours and the local-community-paradigm for topological link prediction in bipartite networks. *New J. Phys.* **2015**, *17*, 113037, doi:10.1088/1367-2630/17/11/113037
13. Durán, C.; Daminelli, S.; Thomas, J.M.; Haupt, V.J.; Schroeder, M.; Cannistraci, C.V. Pioneering topological methods for network-based drug–target prediction by exploiting a brain-network self-organization theory. *Brief. Bioinform.* **2017**, *8*, 3–62.
14. Korgaonkar, M.S.; Fornito, A.; Williams, L.M.; Grieve, S.M. Abnormal structural networks characterize major depressive disorder: A connectome analysis. *Biol. Psychiatry* **2014**, *76*, 567–574.
15. van den Heuvel, M.P.; de Lange, S.C.; Zalesky, A.; Seguin, C.; Yeo, B.T.T.; Schmidt, R. Proportional thresholding in resting-state fMRI functional connectivity networks and consequences for patient-control connectome studies: Issues and recommendations. *Neuroimage* **2017**, *152*, 437–449.
